# Potential of *Pantoea dispersa* as an effective biocontrol agent for black rot in sweet potato

**DOI:** 10.1038/s41598-019-52804-3

**Published:** 2019-11-08

**Authors:** Lingmin Jiang, Jae Chul Jeong, Jung-Sook Lee, Jeong Mee Park, Jung-Wook Yang, Myoung Hui Lee, Seung Hee Choi, Cha Young Kim, Dae-Hyuk Kim, Suk Weon Kim, Jiyoung Lee

**Affiliations:** 1 Korean Collection for Type Cultures (KCTC), Biological Resource Center, Korea Research Institute of Bioscience & Biotechnology (KRIBB), Jeongeup, 56212 Republic of Korea; 2 Plant Systems Engineering Research Center, KRIBB, Daejeon, 34141 Republic of Korea; 3 Crop Cultivation & Environment Research Division, National Institute of Crop Science, Suwon, 16429 Republic of Korea; 4 Department of Bioactive Materials, Chonbuk National University, Jeonju, 54896 Republic of Korea

**Keywords:** Biotic, Microbe

## Abstract

Biocontrol offers a promising alternative to synthetic fungicides for the control of a variety of pre- and post-harvest diseases of crops. Black rot, which is caused by the pathogenic fungus *Ceratocytis fimbriata*, is the most destructive post-harvest disease of sweet potato, but little is currently known about potential biocontrol agents for this fungus. Here, we isolated several microorganisms from the tuberous roots and shoots of field-grown sweet potato plants, and analyzed their ribosomal RNA gene sequences. The microorganisms belonging to the genus *Pantoea* made up a major portion of the microbes residing within the sweet potato plants, and fluorescence microscopy showed these microbes colonized the intercellular spaces of the vascular tissue in the sweet potato stems. Four *P*. *dispersa* strains strongly inhibited *C*. *fimbriata* mycelium growth and spore germination, and altered the morphology of the fungal hyphae. The detection of dead *C*. *fimbriata* cells using Evans blue staining suggested that these *P*. *dispersa* strains have fungicidal rather than fungistatic activity. Furthermore, *P*. *dispersa* strains significantly inhibited *C*. *fimbriata* growth on the leaves and tuberous roots of a susceptible sweet potato cultivar (“Yulmi”). These findings suggest that *P*. *dispersa* strains could inhibit black rot in sweet potato plants, highlighting their potential as biocontrol agents.

## Introduction

Endophytes are microorganisms that reside within plant tissues without causing any apparent disease symptoms in their hosts^1–4^. These organisms interact with plants in a variety of ways, affecting all stages of development through to the end of the plant life cycle^5^. Some endophytes promote plant growth, the mechanisms behind which have been extensively studied and include the facilitation of nutrient uptake (e.g., phosphorus), nitrogen fixation for plant use, the sequestration of iron for plants by siderophores, the production of plant hormones [e.g., gibberellins (GAs), indoleacetic acid (IAA), and cytokines], the elimination of ethylene from plants, reduction of the amount of iron available to plant pathogens in the rhizosphere, the synthesis of fungal cell wall-degrading enzymes, and competition with several phytopathogens^6–9^.

Many biotic and abiotic factors influence the structure and function of the bacterial communities in plants–for example, climate change, pesticide treatment, soil type, plant health, and developmental stage^10–14^. In addition, the composition of the root exudates varies between plants and affects the relative abundance of microorganisms near the root^15^. Not only do plants provide nutrients to microorganisms, but some plant species also contain antimicrobial metabolites that are unique to their exudates^16,17^. Therefore, the compounds that are secreted by the roots act as a signal, attracting or repelling microorganisms to the plants^18,19^, and thus regulating the interaction between the roots and soil microorganisms.

Sweet potato [*Ipomoea batatas* (L.)] is one of the most important cultivated crops in the world, with an annual production area of 8.0 million hectares and a total global production of 106,569 million tons^20^. Sweet potato is also a valuable medicinal plant due to its anti-cancer, anti-diabetic, anti-oxidant, and anti-inflammatory activities^21–23^. However, sweet potato plants experience many post-harvest diseases during transportation and storage. Black rot, which is caused by *Ceratocystis fimbriata*, is one of the most devastating, causing serious economic and resource losses worldwide^24–26^. These pathogenic fungi are carried in the tuberous roots and cause disease in the progeny, and cannot currently be controlled by fungicides or other chemicals. Therefore, the control of black rot in sweet potato is a serious challenge, irrespective of the reproduction, planting, and storage techniques that are used, due to the extensive and intractable nature of this disease^24,26,27^. It has recently been shown that an endophytic bacterium of *Arabidopsis thaliana* known as *Rhodococcus* sp. KB6 inhibits black rot disease caused by *C. fimbriata* in sweet potato leaves^25^. However, there are still few reports of methods for controlling this disease.

A number of endophytic bacteria have been isolated from sweet potato at different production stages and from a range of tissues and plant genotypes^28^. In particular, bacteria in the genus *Bacillus* have been shown to be highly enriched in the tuber rhizosphere of many genotypes of sweet potato, while other genera exhibit a plant genotype dependent affluence^28,29^. In particular, the genera *Brucella*, *Sphingobium*, *Comamonas*, *Methylophilus*, *Pantoea*, *Acinetobacter*, *Pseudomonas*, *Stenotrophomonas*, *Chryseobacterium*, and *Sphingobacterium* are abundant in the tuber rhizosphere of the IPB-137 genotype^28^. Some of the yellow-pigmented, gram-negative bacteria in the genus *Pantoea* (family *Erwiniaceae*) produce antimicrobials and have been developed into commercial biocontrol products to help control fire blight on apple and pear trees, such as BlightBan C9-1 and Bloomtime^TM^ biological^30–32^, while others have bioremediation potential, with the ability to degrade herbicides without generating toxic products^33^. However, while the endophyte *P. agglomerans* has been isolated from the stem of sweet potato^34^, it remains unknown whether bacteria in this genus can counteract the growth of sweet potato fungal pathogens.

In this study, we isolated microorganisms from the shoots and tuberous roots of sweet potato plants growing in the field and examined their inhibition activity against the fungal pathogen *C. fimbriata*. We found that four *P. dispersa* strains (RO-18, RO-20, RO-21, and SO-13) showed strong inhibition activity against *C. fimbriata*, with cell-free culture supernatant inhibiting spore germination and causing cellular changes in the hyphal morphology, including hyphal swelling, distortion, and cytoplasmic aggregation. Furthermore, we confirmed that these bacteria exhibit fungicidal rather than fungistatic activity. These results indicate the value of *P. dispersa* strains for reducing black rot infection in sweet potato and decreasing the use of agricultural chemicals.

## Results

### Molecular identification and phylogenetic analysis of the microbial communities in sweet potato plants

We collected a total of 75 species of microorganisms from the tuberous roots (RO) and shoots (SH) of field-grown sweet potato plants and the bulk soil (SO). These isolates could be identified to the genus and species level with a sequence similarity of 97–100% based on 16S or 5.8S rRNA gene sequences in the NCBI database.

**Figure 1:**
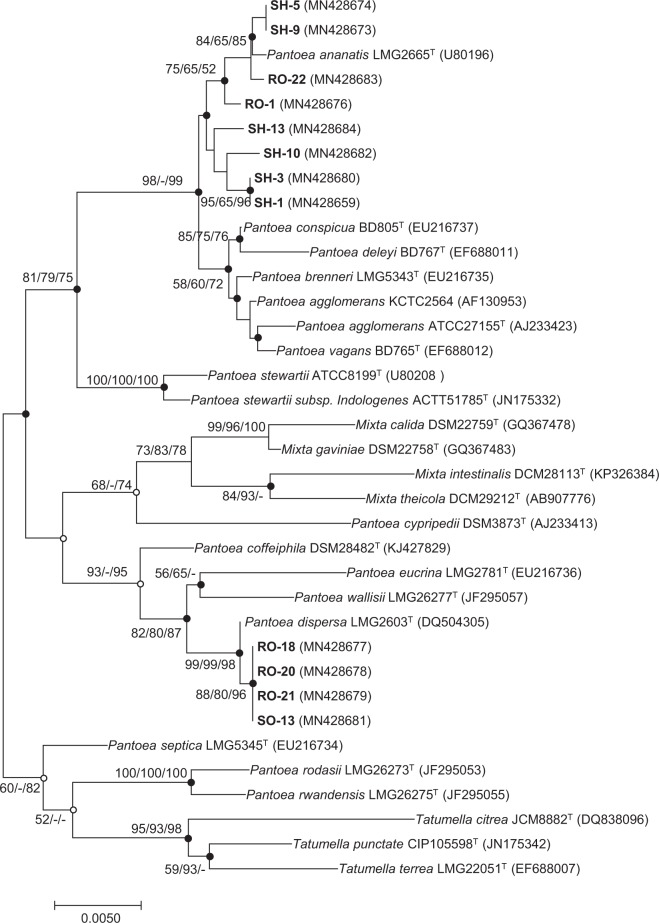
Neighbor-joining phylogenetic tree based on 16S rRNA showing the relative positions of the *Pantoea* isolates and other *Pantoea* type strains. Evolutionary analyses were conducted in MEGA7.0 and bootstrap values (based on 1,000 replicates) greater than 50 are indicated at the branch nodes. Filled circles on the nodes indicate that the relationships were also recovered by ML and ME algorithms, whereas open circles indicate nodes recovered by either the ML or the ME algorithm. The bar represents 0.005 substitutions per nucleotide position.

The isolates were identified as bacteria belonging to 15 genera: *Arthrobacter*, *Bacillus*, *Bacterium*, *Burkholderia*, *Cupriavidus*, *Enterobacter*, *Leclercia*, *Lysinibacillus*, *Microbacteriaceae*, *Microbacterium*, *Pantoea*, *Pseudomonas*, *Psychrobacillus*, *Serratia*, and *Streptomyces*; fungi belong to 17 genera: *Aspergillus*, *Cladosporium*, *Cryptococcus*, *Fusarium*, *Gongronella*, *Mortierella*, *Mucor*, *Neurospora*, *Papiliotrema*, *Penicillium*, *Phoma*, *Rhizomucor*, *Rhodosporidium*, *Rhodotorula*, *Sakaguchia*, *Torula*, and *Mucor*. Results revealed that 75 species endophytic microorganisms were belonging to 32 genera. At the genus level, the samples were predominantly populated with variable numbers of individuals in the genera *Pantoea* and *Bacillus* (Supplementary Tables S2–S4). The phylogenetic tree based on the 16S rRNA, *gyrB* and *rpoB* gene which generated from NJ, ML and ME algorithm showed that all species of the genus *Pantoeas* divided into multiple clades, while *Pantoea* isolates in this study fell into two clades, *P*. *dispersa*, and *P*. *ananatis* (Fig. 1; Supplementary Figs S1 and S2). The isolates RO-18, RO-20, RO-21, and SO-13 were most closely related to the type strain *Pantoea dispersa* LMG2603^T^, while isolates RO-1, RO-22, SH-5, SH-9, SH-1, SH-10, SH-13, and SH-3 were most closely related to the type strain *Pantoea ananatis* LMG2665^T^. These independent monophyletic clades clearly determined the species name of *Pantoea* collected from sweet potato tissue.

### Colonization of *Pantoea* in sweet potato plant tissues

**Figure 2:**
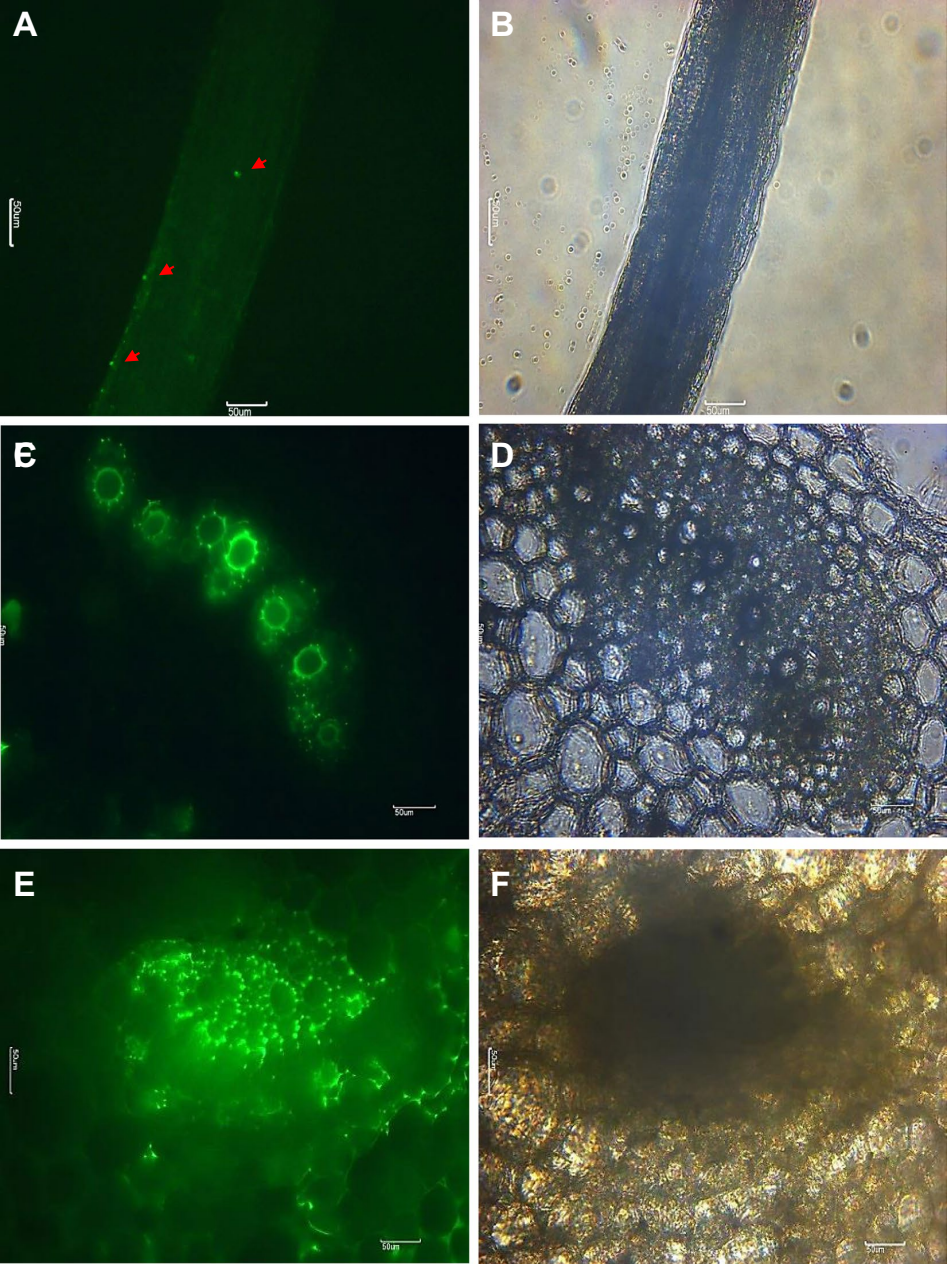
Visualization of green fluorescent protein (GFP)-expressing *Pantoea* in sweet potato (*Ipomoea batatas* “Yulmi”) plant tissues. Sweet potato plants were inoculated with GFP-labeled *P. dispersa* RO-21 at 10^8^ colony-forming units (CFU) mL^−1^. The plant roots and vascular system were then examined under an epifluorescence microscope. (**A**) Single colonies were observed in the roots at 18 h after inoculation. (**C**,**E**) Multiple colonies were observed inside the plant’s vascular system at 48 h after inoculation. (**B**,**D**,**F**) Transmission images of plant cells are shown. Green fluorescence and transmission images were taken at 20× **(A**,**B)** and 40× (**C**–**F**) magnification. Each arrow indicates the position of single colonies of GFP-expressing *Pantoea*. Scale bars are 50 *μ*m.

To visualize the location of *Pantoea* in sweet potato plants by live-cell imaging, we inoculated the roots of the sweet potato cultivar “Yulmi” with a suspension of GFP-labeled *P. dispersa* RO-21. Individual colonies of GFP-expressing RO-21 were observed in the roots 18 h after inoculation (Fig. 2A,B; Supplementary Fig. S3). After 48 h, bacterial aggregates were observed in horizontal sections of stems (Fig. 2C–F), and cells were effectively observed residing in the intercellular spaces of the outer cortex and there was extensive colonization in the cellular pits of the xylem tracheids in the stems (Fig. 2C–F). After 7 d, GFP-expressing macro-colonies of RO-21 was found in the leaf petioles (Supplementary Fig. S4), and it was able to recover by selective medium (Supplementary Fig. S5). This result suggests that *P. dispersa* effectively colonizes sweet potato tissues as an endophytic bacterium.

### Evaluation of *in vitro* antagonism

**Figure 3:**
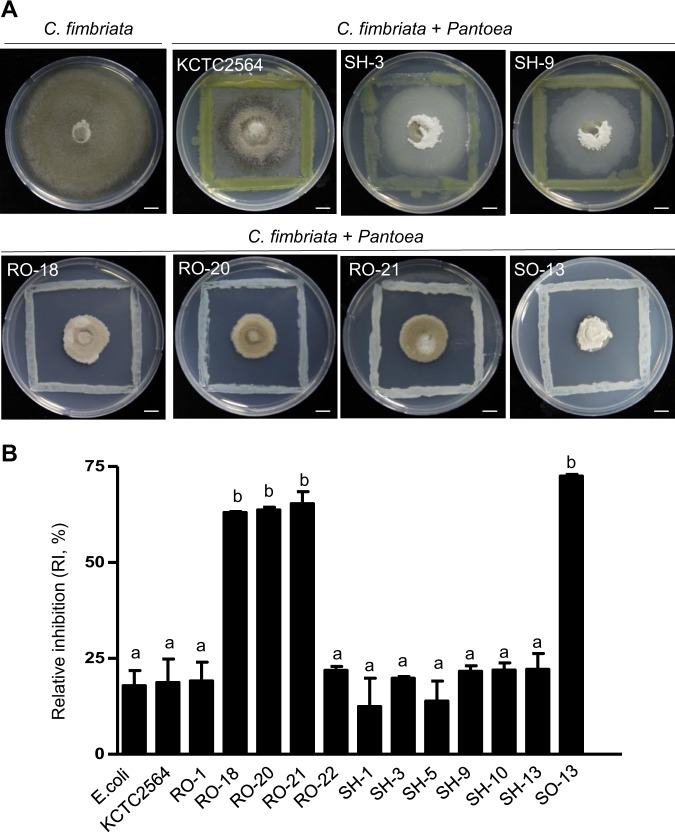
Inhibition effects of *Pantoea* isolates against the phytopathogenic fungus *C. fimbriata*. (**A**) Dual culture assay of *C. fimbriata* and *Pantoea* isolates at 16 d. Agar plugs of *C. fimbriata* (5-mm diameter) were co-incubated with *Pantoea* strains SH-3, SH-9, RO-18, RO-20, RO-21, and SO-13 for 16 d at 28 °C. *Escherichia coli* and *P. agglomerans* KCTC2564 were used as controls (scale bar = 1 cm). (**B**) The relative inhibition (RI) rate of each *Pantoea* strain against *C. fimbrata*. The RI was calculated using the formula RI (%) = [(radial growth in control − radial growth in sample)/radial growth in control] × 100%. The bars indicate the standard errors of triplicate samples. Values followed by a different letter are significantly different (P ≤ 0.05).

To determine the potential of endophytic *Pantoea* to control *C. fimbriata*, *Pantoea* isolates were screened for their inhibitory ability using an *in vitro* dual culture assay on PDA media. Mycelial plugs of *C. fimbriata* (5-mm diameter) were placed at the center of PDA plates and bacterial colonies were streaked around the borders of the plates (Fig. 3A; Supplementary Fig. S6). The antagonistic activity of the *Pantoea* strains was then estimated by comparing the fungal growth with *C. fimbriata* alone. Since *P. agglomerans* was previously identified from sweet potato^31^, we introduced *P. agglomerans* KCTC2564 (=ATCC27155) as additional *Pantoea* strain for this test. Fungal growth was monitored by measuring the diameter of the mycelium until day 16 at 28 °C (Fig. 3A; Supplementary Fig. S6A and S6B). This approach allowed the relative growth of *C. fimbriata* mycelia to be quantitatively assessed (Fig. 3B).

*Pantoea* strains RO-18, RO-20, RO-21, and SO-13, which were identified as *P. dispersa*, had the highest inhibition rates, reaching >63% at 16 d after co-incubation (Fig. 3B). Among these, RO-21 and SO-13 showed the best inhibition activity, decreasing mycelial growth by up to 72% and significantly reducing the diameter of the fungal disks from 7.3 cm with the control treatment to 2.4–2.6 cm (Supplementary Fig. S6B). All four of these strains exhibited a clear inhibition halo (Fig. 3A). Some of the other bacterial strains also showed weak inhibition activity on *C. fimbriata* growth (19–34%), including *Escherichia coli* DH5 *α*, *P. agglomerans* KCTC2564, and *P. ananatis* strains SH-5, SH-9, SH-1, RO-22, SH-3, and RO-1 (Fig. 3A,B; Supplementary Fig. S6A); however, there was no statistically significant reduction in the diameter of the fungal disks with these treatments and the negative control (P ≥ 0.05) (Fig. 3B). These results indicate that *P. dispersa* strains RO-18, RO-20, RO-21, and SO-13 are good candidates as potential biocontrol agents of *C. fimbriata*.

### Effect of cell-free culture supernatant of *Pantoea* on spore germination and hyphal morphology

**Figure 4:**
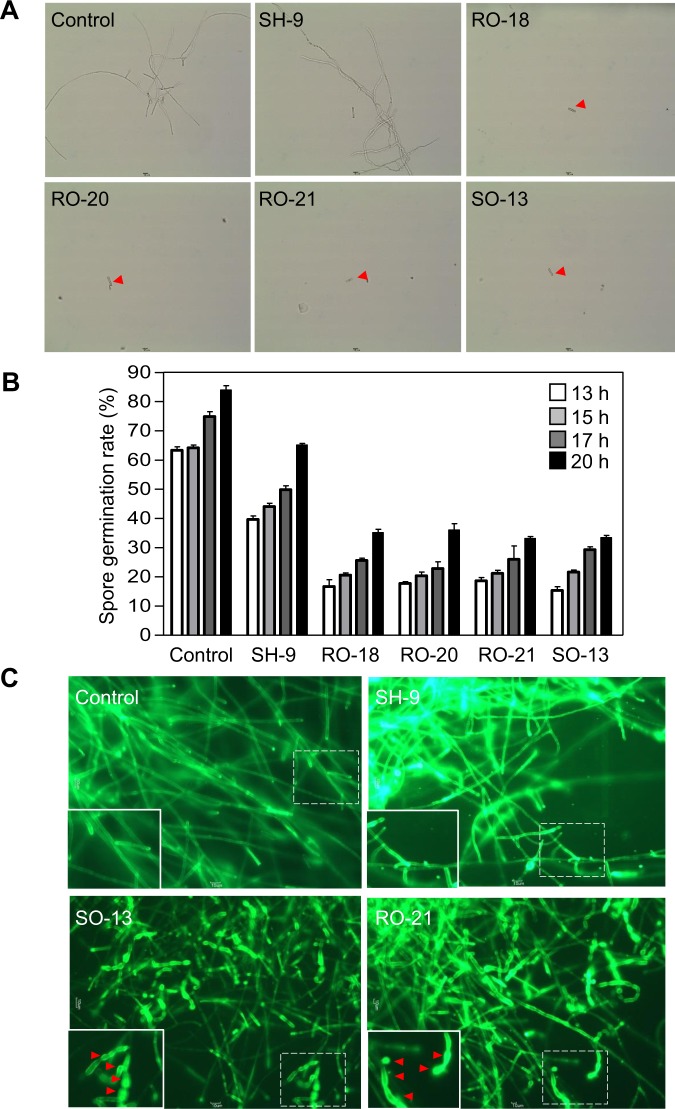
Effects of the cell-free supernatant of *Pantoea* isolates on the *C. fimbriata* spore germination rates and hyphal morphology. (**A**) Effects of various *Pantoea* strains on the *C. fimbriata* spore germination rates. Strains RO-18, RO-20, RO-21, and SO-13 strongly inhibited spore germination, whereas strain SH-9 exhibited only weak antifungal activity and the negative control (no treatment) showed no inhibition. The experiment was repeated twice with similar results. (**B**) Effect of the spore germination rates on the co-culturing time. (**C**) Alteration of the fungal cell wall by cell-free culture supernatant of *Pantoea* isolates. Mycelia were stained with fluorescein isothiocyanate-labeled wheat germ agglutinin (FITC-WGA). Insets show enlarged views of the boxed areas. Arrows indicate hyphal swelling, distortion, and cytoplasmic aggregation.

To determine whether the extracellular compounds that are produced by *P. dispersa* have antifungal activity, we observed the spore germination rates and morphologies of *C. fimbriata* following co–culture of the spores (10^5^ CFU mL^−1^) with 900 *μ*L of cell–free culture supernatant of *Pantoea* strains SH-9, RO-18, RO-20, RO-21, and SO-13. Light microscopy was then used to evaluate the effect of extracellular metabolites in the culture filtrates on the spore germination rates. Incubation with the cell-free culture supernatant of SH-9 did not significantly affect the *C. fimbriata* germination rates compared with the control after 20 h [germination rates = Control (84.0%) and SH-9 (65.1%), respectively]. However, the germination rates of the spores were inhibited by incubation with the cell-free supernatant of RO-18 (35.1%), RO-20 (36.0%), RO-21 (33.1%), and SO-13 (33.4%) after 20 h (Fig. 4A,B). Staining with FITC-WGA further showed that extracellular metabolites in the culture filtrates of *P. dispersa* strains RO-21 and SO-13 caused cellular changes in the hyphal morphology of *C. fimbriata*, including hyphal swelling, distortion, and cytoplasmic aggregation, while incubation with the cell-free culture supernatant of *P. ananatis* strain SH-9 did not cause any changes in hyphal morphology (Fig. 4C). Together, these findings suggest that the extracellular metabolites of *P. dispersa* have antifungal activity against *C. fimbriata*.

### *In vitro* interaction between *Pantoea* isolates and *C. fimbriata*

**Figure 5:**
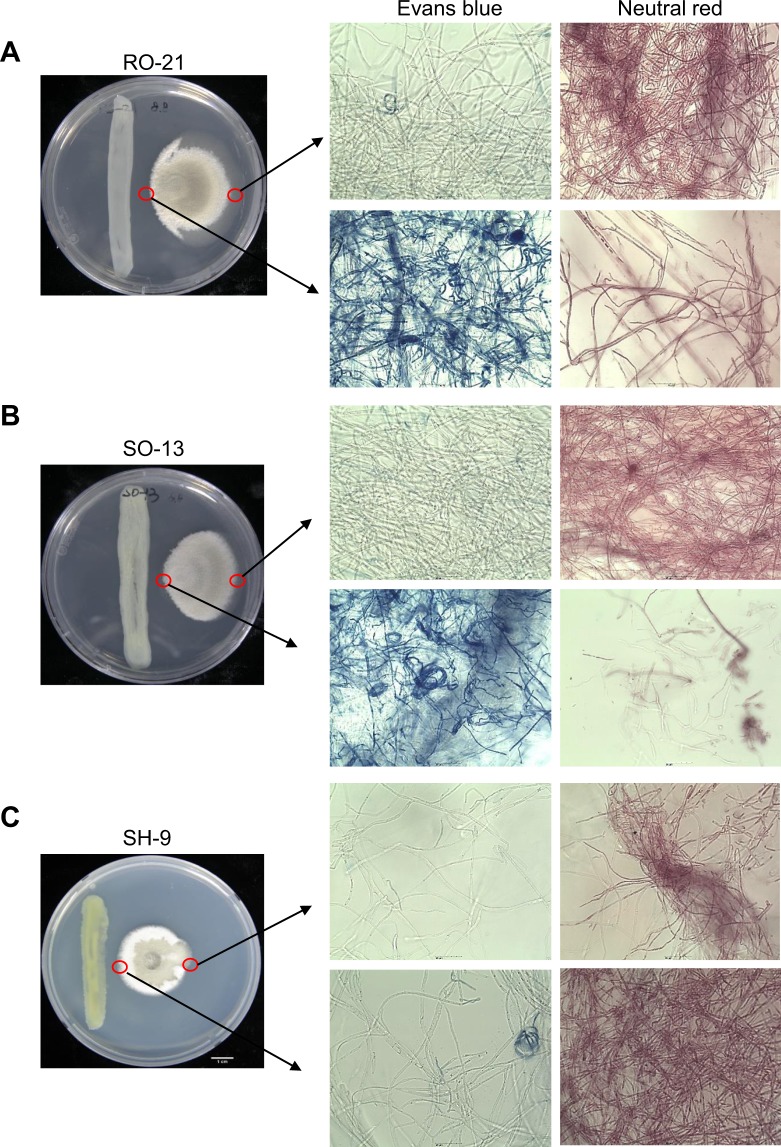
Interaction effect of *C. fimbriata* with *Pantoea* RO-21, SO-13 and SH-9. *C. fimbriata* cells were stained with Evans blue (dead cells stain blue) and Neutral red (viable cells stain red) after co-cultivation with strain RO-21, SO-13 and SH-9 for 10 d. (**A**,**B**) Mycelia growing near to RO-21 and SO-13 stained blue, while those in the control zone on the other side only exhibited faint staining. (**C**) By contrast, mycelia that were co-cultured with SH-9 did not stain blue in any region. Scale bars are 50 *μ*m.

To better understand the mode of action of the antifungal activity of *P. dispersa* strains, *C. fimbriata* was grown alongside *Pantoea* strains and the cell viability was visualized by staining with Evans blue, which stains dead cells blue, and Neutral red, which stains viable cells red. *P. dispersa* RO-21 and SO-13 appeared to cause the dramatic breakage of *C. fimbriata* hyphae in the contact zone, with the mycelia that were exposed to *P. dispersa* staining blue, while those in the control zone on the other side only exhibited faint blue staining (Fig. 5A,B). Similar results were obtained from RO-20 and RO-18 (Supplementary Fig. S7A and S7B). By contrast, in the presence of *P. ananatis* SH-9 and SH-3, faint blue staining and red staining were observed in both the contact and control zones (Fig. 5C; Supplementary Fig. 7C). These findings indicate that *P. dispersa* has fungicidal activity rather than fungistatic activity against *C. fimbriata*.

### Inhibition of *C. fimbriata* infection in sweet potato leaves and tuberous roots

**Figure 6:**
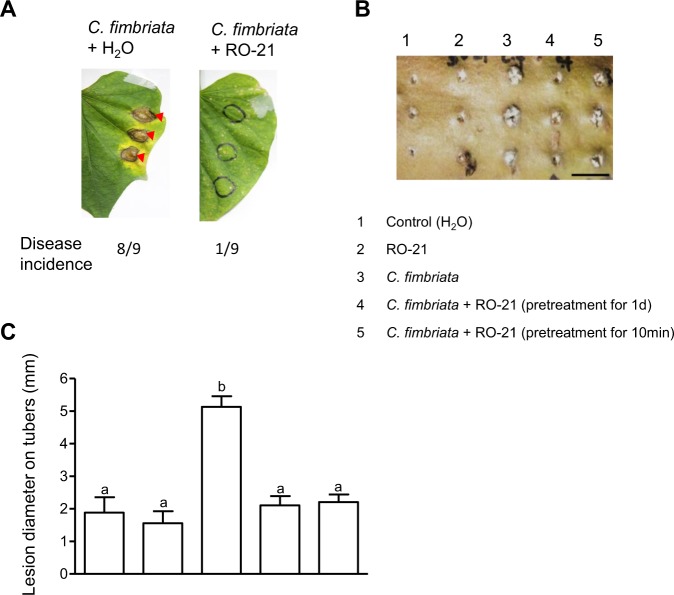
Effects of co-inoculation with *Pantoea* RO-21 and *C. fimbriata* on the detached leaves and tuberous roots of sweet potato (*Ipomoea batatas* “Yulmi”) plants. (**A**) Disease incidence in the leaves of sweet potato plants pre-treated with water (control; left) and RO-21 (right) at 36 h after co-incubation with a spores droplet of *C. fimbriata*. (**B**) Disease incidence in the tuberous roots at 36 h after inoculation following pre-treatment with RO-21 or water (control) at 10 d after co-incubation with a spores droplet of *C. fimbriata* (scale bar = 1 cm). (**C**) Diameter of the lesion region, as evaluated by ImageJ analysis. The values shown are the average of at least two independent experiment performed in triplicate. Error bars indicate the standard error of the means (SEM) values. Values followed by a different letter are significantly different (n = 3–6, P ≤ 0.05).

To assess the biocontrol efficiency of *P. dispersa* in the leaves and tuberous roots of the sweet potato cultivar “Yulmi”, which is susceptible to *C. fimbrata* infection^23^, samples were pre-treated with RO-21 or water (as a control) and then inoculated at the same site with droplets containing *C. fimbriata* spores. The disease incidence on the leaves and tuberous roots was then determined. Leaves that had been pre-treated with water showed disease symptoms at the inoculation site, with dark brown circular patches of 4–6 mm diameter appearing on the upper surface of the leaves and the surrounding areas turning yellow after 36 h. By contrast, the disease incidence was reduced in leaves that had been pre-treated with RO-21 (Fig. 6A). Disease symptoms were also dramatically reduced in tuberous roots that had been pre-treated with RO-21 cells for 1 d before *C. fimbriata* inoculation compared with those that had been pre-treated with water as a control (Fig. 6B,C). Furthermore, pre-treatment with RO-21 for only 10 min also reduced the size of the black lesions around the inoculation site compared with the water pre-treatment (Fig. 6C). Together, these findings indicate that *P. dispersa* could prevent the infection of sweet potato leaves and tuberous roots by the pathogenic fungus *C. fimbriata*.

## Discussion

The composition of any given plant-associated microbial community is likely to be determined by multiple factors, including the soil type, phytopathogen population^12^, plant age^35^, and plant genotype, as well as stochastic sampling factors^36^. In the present study, we identified 75 culturable microbes that were associated with field-grown sweet potato plants based on their colony morphologies, colors, and patterns of growth. These included members of the genera *Bacillus*, *Pantoea*, *Enterobacter*, *Serratia*, and *Microbacterium* (Supplementary Tables S2 and S3), indicating that these plants are associated with diverse bacterial communities. However, only a few species dominated these communities, with the genera *Bacillus* and *Pantoea* predominating in the shoots and tuberous roots (Supplementary Tables S2 and S3).

*Bacillus* species are considered attractive biocontrol agents due to their ability to produce hard, resistant endospores and antibiotics that control a broad range of plant fungal pathogens^37^. However, it has previously been found that *B. subtilis* does not effectively inhibit the growth of *C. fimbriata*^38^, which is consistent with the findings for the *Bacillus* strains isolated in the present study (data not shown). The bacterial genus *Pantoea* comprises many versatile species that have been isolated from a multitude of environments, such as mammals, agricultural areas, water, and particularly soil^39^. El Amraoui^40^ found that species showed stronger antifungal activity against *Candida albicans* CIP 48.72, *Candida albicans* CIP 884.65, and *Cryptococcus neoformans* CIP 960 than *Bacillus* sp., while Town^41^ showed that *Pantoea* sp. inhibits the potato pathogen *Phytophthora infestans*. In the present study, *Pantoea* strains RO-18, RO-20, RO-21, and SO-13, which were isolated from sweet potato, had a high homology with the type strain *P. dispersa* LGM 2603^T^ and exhibited remarkable antifungal activity against *C. fimbriata* both *in vitro* and *in vivo*, with the *in vitro* dual culture assay showing that they inhibited *C. fimbriata* mycelium growth (Fig. 3A) and spore germination (Fig. 4A,B), appeared to cause dramatic breakage of the fungal hyphae (Fig. 4C), and killed the fungal hyphae, acting as a fungicidal agent (Fig. 5A,B). Therefore, genus *Pantoea* has a greater potential to control *C. fimbriata* than genus *Bacillus*.

Root-inoculated GFP-labeled RO-21 cells were initially visualized in the root surface and central vascular system of primary root in sweet potato plants within 18 h, following which a proliferation of cells was observed in the intercellular spaces between adjacent xylem tracheid cells in the caulosphere at 48 h after inoculation. The rapid spread of this strain from the root to the aerial tissues suggests that it uses the vascular system as a route for systemic colonization. Other endophytic bacteria are also located in the vascular system, with large fluorescent colonies having been clearly observed in the pits of the xylem cells in the plant cell wall^42,43^.

The mode of action of *P. dispersa* is similar to that of *B. subtilis* against *Eutypa lata* in grapes, whereby irregularities occur in the hyphal morphology, such as tip narrowing and vesicle formation^44^. *B. subtilis* has been shown to cause abnormalities in the hyphae of *Fusarium oxysporum*, causing cell wall lysis, breakage, granulation, and vacuolization^45^. Similar phenomenon was observed by *P. dispersa* isolates (Figs 4 and 5). Moreover, the leaves and tuberous roots of sweet potato plants had a significantly decreased incidence of disease symptoms when cultured in the presence of RO-21 (Fig. 6A,B), indicating that *P. dispersa* can inhibit the development of black rot disease in sweet potato. Moreover, strains RO-18, RO-20, RO-21, and SO-13 were initially isolated from the tuberous roots of agricultural sweet potato and soil, which can be explained by the fact that both *P. dispersa* and *C. fimbriata* are soil-borne microorganisms that primarily compete for the tuberous roots^5^.

Although *P. ananatis* strains SH-1, SH-3, SH-5, SH-9, SH-10, SH-13, RO-1, and RO-22 did not effectively inhibit the growth of *C. fimbriata* in this study, it has previously been shown that *P. ananatis* CPA-3 has strong antifungal activity against *Penicillium expansum*^46^. Similarly, *P. ananatis* 125NP12 have been found to protect tomato fruit against the grey mold fungus, *Botrytis cinerea*, by producing antifungal compounds^47^. In this respect, *P. ananatis* strains SH-1, SH-3, SH-5, SH-9, SH-10, SH-13, RO-1, and RO-22 might control other plant pathogens in sweet potato plants. In addition, the potential of *P. ananatis* SCB4789F-1 to promote plant growth has been demonstrated by its ability to solubilize phosphorus and zinc, produce siderophores, and synthesize IAA. *P. ananatis* B1-9 strain isolated from the rhizosphere of onions showed potential for promoting plant growth in peppers, cucumbers, and melons^48^. Therefore, further detailed studies are needed on the plant growth-promoting effect of *P. ananatis* isolates.

Several mechanisms have been reported for the biocontrol of plant pathogens, including competition for nutrients^49^, the induction of host resistance^50,51^, and the production of killer toxins^52^, degradable enzymes such as chitinase^6^, and antifungal metabolites^53–55^. Many *Pantoea* strains are strong environmental competitors that produce a variety of natural products with antibiotic activity, such as pantocins, microcins, and phenazines^56–64^. More recently, *Pantoea* Natural Product 1 (PNP-1), which has been isolated from *P. ananatis* BRT175, has been shown to have inhibitory activity against *Erwinia amylovora* and is likely to be similar in action to 4-formylaminooxyvinylglycine (FVG)^65,66^. In the present study, cell-free supernatant derived from *P. dispersa* cultures was found to have inhibitory effects on *C. fimbriata* spore germination and hyphae growth when added simultaneously or after some time, indicating that the antifungal activity of these strains may occur via similar mechanisms. Hence, further investigation is required to evaluate the potential bioactive compounds that may be of biocontrol importance in the metabolites of these isolates.

Together, our results indicate that *P. dispersa* strains represent useful biocontrol agents for protecting sweet potato plants from post-harvest infection by *C. fimbriata* and for decreasing the use of agricultural chemical.

## Methods

### Isolation of microorganisms

Tuberous roots and shoots were collected from healthy sweet potato (*I. batatas*) plants growing in a cultivation area in Jeongeup, Jeollabuk-do, Republic of Korea (35°30′29.8″N, 126° 50′13.9″E). To acquire plant extracts, the plant materials were extensively washed with sterile distilled water, and aliquot (100 *μ*L) of the final rinse water was plated onto Laurie-Bertani (LB) and Potato Dextrose agar (PDA) media (Difco) to check the disinfection process. The plant tissues were then crushed with 50 mL of sterile phosphate-buffered saline (PBS: 100 mM Tris-HCl, pH 8.0; 150 mM NaCl) and filtered through four layers of sterile cheesecloth. The bacterial cells in the filtrate were serially diluted with PBS buffer, and 100 *μ*L of each diluted sample was spread onto an LB and PDA media plates, and then incubated at 28 °C for 5–14 d.

The colony types in each sample were categorized according to their growth rate and phenotypic characteristics, such as sizes, colors, and colony morphology. Each type was then counted and a representative was taken for purification and identification. The following voucher specimens were deposited at the Korean Collection for Type Cultures (KCTC), Korea Research Institute of Bioscience & Biotechnology (KRIBB): KCTC 62154 (SO-13), KCTC 62153 (SH-13), KCTC 62152 (SH-10), KCTC 62151 (SH-9), KCTC 62150 (SH-5), KCTC 62149 (SH-3), KCTC 62148 (SH-1), KCTC 62147 (RO-22), KCTC 62146 (RO-21), KCTC 62145 (RO-20), KCTC 62144 (RO-18), and KCTC 62143 (RO-1).

### Species identification and phylogenetic analysis using 16S and 5.8S rRNA gene sequences

The 16S, 5.8S rRNA and housekeeping genes (*gyrB*, and *rpoB*)^67^ of each species were amplified using the primers listed in Supplementary Table S1. 16S and 5.8S rRNA sequencing was carried out at BioFact Inc. (Daejeon, Korea), and sequence alignment was performed with the BLAST search (https://blast.ncbi.nlm.nih.gov/Blast.cgi). Phylogenetic trees were constructed using the neighbor-joining (NJ), minimum-evolution (ME), and maximum-likelihood (ML) method based on 16S, *gyrB*, and *rpoB* genes in MEGA (ver.7)^68^, and a bootstrap analysis of 1,000 replications was performed to evaluate the stability of the nodes.

### Introduction of a plasmid-born green fluorescent protein (GFP) reporter into *P. dispersa* RO-21

To obtain GFP-expressing *P. dispersa* RO-21, the bacterial cells were transformed with the plasmid pGFPuv^TM^ (Clontech Laboratories Inc., CA, USA). 1 *μ*L of the plasmid (200 ng *μ*L^−1^) was introduced into the cells by electroporation with the Gene Pulse Xcell^TM^ system (Bio-Rad, Hercules, CA, USA) in a 0.2-cm electroporation cuvette (2500 V cm^−1^, 25 *μ*F, 200 Ω). The cells were allowed to recover in 1 mL of LB broth and then incubated for 30 min at 30 °C with shaking (150 rpm). The cells were then spread on a LB plate with ampicillin (100 *μ*g mL^−1^) and incubated at 30 °C overnight to select the transformants, which were identified using ultraviolet (UV) light.

### Monitoring bacterial colonization on sweet potato plants

Sweet potato plants that had been separated from their tuberous roots were placed in a continuous-floating hydroponic system for 5 d until their roots had reached a length of 4–5 cm. The roots of the plants were then inoculated with a suspension of GFP-expressing *P. dispersa* RO-21 [10^8^ colony-forming units (CFU) mL^−1^]. After 15 min, the plant roots were washed three times with distilled water and then kept in the floating hydroponic system. GFP-expressing bacteria were detected in the root 18 h after inoculation. At 4 d and 7 d after inoculation, the stems of each sweet potato plants were cut into sections that were as thin as possible using a razor blade from 5 cm above the roots. Stem cross-sections and selected roots from each plant were then placed on a glass slide with distilled water, and the GFP-expressing bacteria were observed using an epifluorescence microscope with a GFP filter (Moticam Pro 205 A; Motic) at 20× or 40× magnification. To confirm the GFP signals were not due to the auto-fluorescence of the plant tissue itself, re-isolation of the *Pantoea* strain RO-21 were performed. Leaf petioles from the strain RO-21 inoculated plants for 7 d were collected, then surface-sterilized by using 1.05% sodium hypochlorite for 10 min and several repeats rinse in sterile distilled water. After homogenized with PBS buffer, samples were spread on LB agar plates supplementary with ampicillin (100 *μ*g mL^−1^).

### Dual culture assay

To test the activity of the bacterial strains against *C. fimbriata*, mycelial plugs of *C. fimbriata* (5-mm diameter) were placed in the center of PDA plates (pH 5.7) and the various bacterial strains were streaked in a square shape around each agar disk at a distance of 3 cm from the mycelial plug. In addition, mycelial plugs were placed on PDA plates without any bacterial strains as a negative control. Fungal growth was assessed by measuring the diameter (in centimeters) of the colony until 16 d at 25 °C. Each bacterial strain was tested in triplicate and the experiment was carried out twice. The relative inhibition (RI) was then calculated using the formula RI (%) = [(radial growth in control − radial growth in samples)/radial growth in control] × 100, as previously described^69^. Data were analyzed by one-way analysis of variance (ANOVA) using GraphPad Prism 4. Significant differences (P ≤ 0.05) between the means were determined by unpaired t-test and Tukey’s multiple comparison test.

### Microscopic analysis

A fungal spore germination assay was performed using cell-free supernatant of the *Pantoea* strains. Bacterial cells were harvested from 6-d-old liquid cultures of SH-9, RO-18, RO-20, RO-21, and SO-13 by centrifugation for 20 min at 10,000×g. The resulting supernatant was then filtered through a 0.2 *μ*m filter (Whatman^TM^) to remove the cells. *C. fimbriata* spores were collected from a 2-week-old culture of fungi growing on PDA media and placed in 10 mL of sterile distilled water, following which they were passed through 25 *μ*m sterile Miracloth^TM^ (Millipore) to remove the hyphae and subsequently diluted to 10^5^ spore mL^−1^ for the bioassay. Then, 10^4^ spores were co-incubated with 900 *μ*L of each of the cell-free supernatant of SH-9, RO-18, RO-20, RO-21, and SO-13 for 13, 15, 17, and 20 h in triplicate. Spore germination (%) was evaluated in each sample by placing the spores on a microscope slide and counting at least 300 spores per sample. Once the fungal spores had been left to germinate in the cell-free supernatant of the *Pantoea* strains for 36 h, the hyphae were stained with fluorescein isothiocyanate-labeled wheat germ agglutinin (FITC-WGA, 1 *μ*g mL^−1^) for 30 min to investigate whether the cell-free supernatant of *P. dispersa* altered the fungal cell wall.

To evaluate the viability of this pathogenic fungus in the presence of *Pantoea* strains, *C. fimbriata* was co-cultivated alongside RO-18, RO-20, RO-21, SH-3 or SH-9 on PDA media at 25 °C for 10 d. The *C. fimbriata* mycelia that were adjacent to the bacteria, were then stained with the vitality stains Neutral red (0.1 mg mL^−1^; Dae Jung, Cat # 5603-4125) or Evans blue (0.5 mg mL^−1^; Alfa Aesar, Cat # A16774) by placing 10 *μ*L of the solution on a slide, incubating the cells for 3–5 min at room temperature, and then washing 3–4 times with deionized water. Mycelia growing on the opposite sides from the bacteria were treated as a negative control. There were 3–4 replicates per treatment and photographs were taken under a light microscope (Nikon Eclipse Ci).

### Disease incidence and symptoms

To evaluate the biocontrol effect of *P. dispersa* on *C. fimbriata*, the leaves (5–6 weeks old) and tuberous roots of plants of the sweet potato cultivar “Yulmi”, which is susceptible to *C. fimbrata*, were analyzed. The leaf surfaces were punched with a needle on the upper side, and then the punched sites were inoculated with 10 *μ*L of *P. dispersa* cell suspension (10^7^ CFU mL^−1^) pre-treatment for 1 d. The *C. fimbriata* spores after incubation for 7 d at 25 °C were scraped from well-grown PDA plates and filtered through 25 *μ*m Miracloth in order to obtain spore suspensions. 5 *μ*L of *C. fimbriata* suspension (10^6^ CFU mL^−1^) were inoculated at the same site as used for pre-treatment. The inoculated leaves were covered with plastic wrap for 36 h, the yellow to black area of the punched leaves were recorded. All experiments were performed in triplicate, repeated at least twice. Surface of the tuberous roots was punched with a needle, then same treatment as described with leaves, unless inoculated with 10 *μ*L of *P. dispersa* cell suspension (10^7^ CFU mL^−1^) pre-treatment for 1 d and 10 min, then inoculated with 5 *μ*L of *C. fimbriata* suspension at the same site as was used for pre-treatment, under moist conditions in the plastic box at 25 °C for 10 d. The disease incidence was recorded by observing the formation of the black spots of *C. fimbriata* and softening of the tissue in the collar region. All measurements were made in triplicate and repeated twice with similar results.

## Supplementary information

Supplementary Dataset
