# Supplementary material for: Potential of Pantoea dispersa as an effective biocontrol agent for black rot in sweet potato
Source: Sci Rep. 2019 Nov 8;9:16354. doi: 10.1038/s41598-019-52804-3 (PMC6841936; doi:10.1038/s41598-019-52804-3)
Supplement: Supplementary file 1 — Supplementary Dataset [file 41598_2019_52804_MOESM1_ESM.pdf]

Supplementary Information

Potential of *Pantoea dispersa* as an effective biocontrol agent for black rot in sweet potato

Lingmin Jiang[1,4] • Jae Chul Jeong[1] • Jung-Sook Lee[1] • Jeong Mee Park[2] • Jung-Wook Yang[3] • Myoung Hui Lee[1] • Seung Hee Choi[1] • Cha Young Kim[1] • Dae-Hyuk Kim[4] • Suk Weon Kim[1,*] • Jiyoung Lee[1,*]

[1]Korean Collection for Type Cultures (KCTC), Biological Resource Center, Korea Research Institute of Bioscience & Biotechnology (KRIBB), Jeongeup, 56212, Republic of Korea

[2]Plant Systems Engineering Research Center, KRIBB, Daejeon,34141, Republic of Korea

[3]Crop Cultivation & Environment Research Division, NICS, Suwon, 16429, Republic of Korea

[4]Department of Bioactive Materials, Chonbuk National University, Jeonju, 54896 Republic of Korea

*Corresponding authors: Suk Weon Kim and Jiyoung Lee

Email: (SWK) kimsw@kribb.re.kr; (JL) jiyoung1@kribb.re.kr

Supplementary Table S1. The primers that were used in this study

Primer name	Target	Sequence (5'→3')	No. bases
518 F	16S rRNA	CCA GCA GCC GCG GTA ATA C	19
805 R	16S rRNA	GAC TAC CAG GGT ATC TAA TC	20
21 F	16S rRNA	TTC CGG TTG ATC CYG CCG GA	20
1492 R	16S rRNA	TAC GGY TAC CTT GTT ACG ACT T	22
ITS1	5.8S rRNA	TCC GTA GGT GAA CCT GCG G	19
ITS4	5.8S rRNA	TCC TCC GCT TAT TGA TAT GC	20
gyrB3	*gyrB*	GCG TAA GCG CCC GGG TAT GTA	21
gyrB4	*gyrB*	CCG TCG ACG TCC GCA TCG GTC AT	23
gyrB3iseq	*gyrB*	AAC GCW ATC GAC GAA GC	17
gyrB4iseq	*gyrB*	TGG AAV CCR TCR TTC CAC	18
Vic3	*rpoB*	GGC GAA ATG GCW GAG AAC CA	20
Vic2	*rpoB*	GAG TCT TCG AAG TTG TAA CC	20

Supplementary Table S2. Molecular identification of the microorganisms that were isolated from sweet potato tuberous roots

Isolate	Accession no.	Tissue	16S/5.8S rRNA (bp)	Closest species in Genbank	Identity
RO-1	MN428676	Tuberous roots	1341	*Pantoea* sp. XFZ20	99%
RO-3	MN524106	Tuberous roots	1448	*Enterobacter kobei* DSM 13645	99%
RO-4	MN521826	Tuberous roots	409	*Papiliotrema laurentii*	100%
RO-5	MN524107	Tuberous roots	1458	*Bacillus toyonensis* PK5-10	99%
RO-6	MN525288	Tuberous roots	592	*Neurospora crassa*	99%
RO-7	MN519610	Tuberous roots	1431	*Leclercia adecarboxylata*	99%
RO-8	MN525289	Tuberous roots	537	*Sakaguchia dacryoidea* AFTOL-ID	98%
RO-11	MN521827	Tuberous roots	672	*Gongronella butleri* JGS01	99%
RO-12	MN521828	Tuberous roots	579	*Rhodosporidium toruloides*	99%
RO-13	MN524109	Tuberous roots	1405	*Bacillus safensis* SL-40	100%
RO-14	MN524110	Tuberous roots	1403	*Bacillus aerius* JS-786	100%
RO-15	MN524111	Tuberous roots	1417	*Bacillus* sp. FJAT-25810	100%
RO-16	MN524112	Tuberous roots	1415	*Bacillus* sp. NT5	100%
RO-17	MN524113	Tuberous roots	1413	*Bacillus megaterium*	100%
RO-18	MN428677	Tuberous roots	1467	*Pantoea dispersa* GTC 1472	99%
RO-19	MN524114	Tuberous roots	1415	*Bacillus aerius* 24K	99%
RO-20	MN428678	Tuberous roots	1403	*Pantoea dispersa* S10	100%
RO-21	MN428679	Tuberous roots	1415	*Pantoea dispersa* strain DSM 30073	99%
RO-22	MN428683	Tuberous roots	1253	*Pantoea* sp. XBGRY4	98%
RO-23	MN524115	Tuberous roots	1411	*Bacillus subtilis* F6-2-31	100%
RO-24	MN524116	Tuberous roots	1402	*Bacillus thuringiensis* FJAT-28888	100%
RO-25	MN524108	Tuberous roots	1389	*Enterobacter aerogenes* QTYC24b	100%
RO-26	MN524128	Tuberous roots	1408	*Serratia marcescens* FZSF01	100%
RO-27	MN524117	Tuberous roots	1444	*Bacillus subtilis* CICC 10366	99%

Supplementary Table S3. Molecular identification of the microorganisms that were isolated from sweet potato shoots

Isolate	Accession no.	Tissue	16S/5.8S rRNA (bp)	Closest species in Genbank	Identity
SH-1	MN428659	Shoot	1391	*Pantoea ananatis* PP1	99%
SH-3	MN428680	Shoot	1411	*Pantoea ananatis* LMG 20103	99%
SH-4	MN521829	Shoot	609	*Rhodotorula* sp. CBS 10104	99%
SH-5	MN428674	Shoot	1401	*Pantoea ananatis* YJ76	99%
SH-6	MN521814	Shoot	508	*Mucor circinelloides* f. *lusitanicus*	100%
SH-8	MN521815	Shoot	600	*Mucor irregularis* CMFCCC B 50r	99%
SH-9	MN428673	Shoot	1394	*Pantoea* sp. BT-1	99%
SH-10	MN428682	Shoot	1408	*Pantoea agglomerans* DSM 3493	98%
SH-11	MN524118	Shoot	1403	*Curtobacterium citreum* H00109	99%
SH-12	MN521817	Shoot	526	*Fusarium fujikuroi* MF22418	99%
SH-13	MN428684	Shoot	1401	*Pantoea ananatis* AJ13355	99%
SH-14	MN519611	Shoot	1386	*Microbacterium* sp. HBUM178903	100%
SH-16	MN521812	Shoot	633	*Rhizomucor variabilis* CBS 103.93	98%

Supplementary Table S4. Molecular identification of the microorganisms that were isolated from the soil

Isolate	Accession no.	Tissue	16S/5.8S rRNA (bp)	Closest species in Genbank	Identity
SO-1	MN525287	Soil	616	*Papiliotrema laurentii* JCM 28239	99%
SO-2	MN521811	Soil	519	*Cryptococcus laurentii* RY1	100%
SO-3	MN524129	Soil	1468	*Serratia marcescens* strain UMH12	99%
SO-4	MN521813	Soil	617	*Mortierella* sp. TR065	99%
SO-5	MN521816	Soil	615	*Mucor circinelloides* f. *lusitanicus*	100%
SO-6	MN519612	Soil	1456	*Burkholderia lata* FL-1-2-30-S1-D0	100%
SO-7	MN519613	Soil	948	*Pseudomonas fluorescens* CLW17	99%
SO-8	MN524119	Soil	1402	*Bacillus safensis* ADU20	100%
SO-9	MN519614	Soil	1413	*Bacterium* CDSHDTYG-6	100%
SO-10	MN524120	Soil	1409	*Bacillus aerius* JS-786	100%
SO-11	MN524121	Soil	1401	*Bacillus* sp. Suaeda B-003	100%
SO-12	MN519615	Soil	1399	*Psychrobacillus psychrodurans* NB-9	100%
SO-13	MN428681	Soil	1215	*Pantoea dispersa* Y08	100%
SO-14	MN519616	Soil	1385	*Arthrobacter* sp. THG-HS1V	99%
SO-15	MN524122	Soil	1469	*Bacillus* sp. Ag1.10	99%
SO-16	MN524123	Soil	1410	*Bacillus* sp. Suaeda B-003	100%
SO-17	MN519617	Soil	1104	*Lysinibacillus* sp. KR25	97%
SO-18	MN524124	Soil	1402	*Bacillus* sp. NT4	100%
SO-19	MN524125	Soil	1409	*Lysinibacillus xylanilyticus* FJAT-29968	99%
SO-20	MN519618	Soil	1369	*Arthrobacter* sp. THG-HS1V	99%
SO-21	MN519619	Soil	1437	*Pseudomonas fluorescens* FW300	100%
SO-22	MN519620	Soil	1391	*Pseudomonas fluorescens*	99%
SO-23	MN521820	Soil	541	*Torula herbarum* isolate G10	100%
SO-25	MN521821	Soil	532	*Phoma herbarum* G10	99%
SO-26	MN521822	Soil	587	*Penicillium* sp. 196F	99%
SO-27	MN519621	Soil	1431	*Cupriavidus* sp. TBSe5	99%
SO-28	MN521823	Soil	569	*Penicillium* sp. BE	100%
SO-29	MN519622	Soil	1429	*Microbacteriaceae bacterium* YR2-6	99%
SO-30	MN519711	Soil	537	*Ascomycota* sp. isolate SK2YWS-L	100%
SO-31	MN521824	Soil	587	*Penicillium* sp. 196F	99%
SO-34	MN524126	Soil	1436	*Bacillus thuringiensis* L14	99%
SO-35	MN521825	Soil	582	*Penicillium* sp. YZ02	99%
SO-36	MN521810	Soil	571	*Aspergillus carneus* W2-4	100%
SO-37	MN519623	Soil	1451	*Streptomyces* sp. Je36-6	99%
SO-38	MN524127	Soil	1442	*Bacillus subtilis* ZD-16	100%
SO-39	MN521809	Soil	533	*Cladosporium subuliforme* INIFAT	100%
SO-40	MN521818	Soil	533	*Aspergillus chevalieri* SRW9 18S	100%
SO-41	MN521819	Soil	569	*Aspergillus versicolor* WZ-2008-0062	100%

Fig. S1

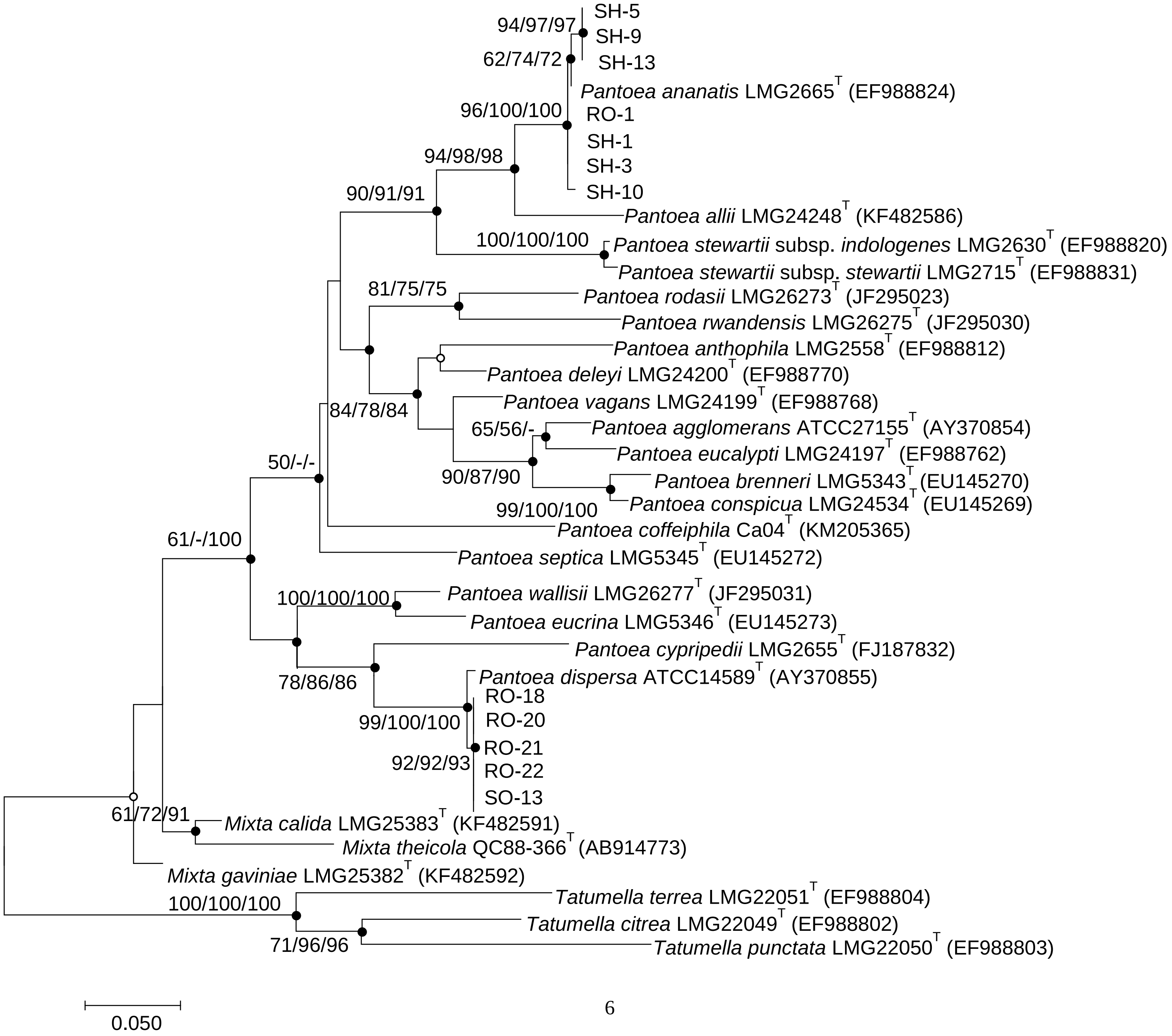
SH-5
SH-9
SH-13
Pantoea ananatis LMG2665T (EF988824)
RO-1
SH-1
SH-3
SH-10
Pantoea allii LMG24248T (KF482586)
Pantoea stewartii subsp. indologenes LMG2630T (EF988820)
Pantoea stewartii subsp. stewartii LMG2715T (EF988831)
Pantoea rodasii LMG26273T (JF295023)
Pantoea rwandensis LMG26275T (JF295030)
Pantoea anthophila LMG2558T (EF988812)
Pantoea deleyi LMG24200T (EF988770)
Pantoea vagans LMG24199T (EF988768)
Pantoea agglomerans ATCC27155T (AY370854)
Pantoea eucalypti LMG24197T (EF988762)
Pantoea brenneri LMG5343T (EU145270)
Pantoea conspicua LMG24534T (EU145269)
Pantoea coffeiphila Ca04T (KM205365)
Pantoea septica LMG5345T (EU145272)
Pantoea wallisii LMG26277T (JF295031)
Pantoea eucrina LMG5346T (EU145273)
Pantoea cypripedii LMG2655T (FJ187832)
Pantoea dispersa ATCC14589T (AY370855)
RO-18
RO-20
RO-21
RO-22
SO-13
Mixta calida LMG25383T (KF482591)
Mixta theicola QC88-366T (AB914773)
Mixta gaviniae LMG25382T (KF482592)
Tatumella terrea LMG22051T (EF988804)
Tatumella citrea LMG22049T (EF988802)
Tatumella punctata LMG22050T (EF988803)
94/97/97
62/74/72
96/100/100
94/98/98
90/91/91
100/100/100
81/75/75
84/78/84
65/56/-
90/87/90
99/100/100
50/-/-
61/-/100
100/100/100
78/86/86
99/100/100
92/92/93
61/72/91
100/100/100
71/96/96
0.050

Supplementary Fig. S1. Phylogenetic analysis using the maximum-likelihood (ML) algorithm based on the *gyrB* gene of *Pantoea* isolates and all the *Pantoea* type strains. Bootstrap values (>50%) were calculated using the Neighbor-joining (NJ), and minimum-evolution (ME) algorithm probabilities. Filled circles on the nodes indicate that the relationships were also recovered by NJ and ME algorithms, whereas open circles indicate nodes recovered by either the NJ or the ME algorithm. Bootstrap values are shown on the nodes in percentages of 1000 replications only for values over 50%. The scale bar equals 0.050 changes per nucleotide position.

Supplementary Fig. S2. Phylogenetic analysis using the maximum-likelihood (ML) algorithm based on the *rpoB* gene of *Pantoea* isolates and all the *Pantoea* type strains. Bootstrap values (>50%) were calculated using the Neighbor-joining (NJ), and minimum-evolution (ME) algorithm probabilities. Filled circles on the nodes indicate that the relationships were also recovered by NJ and ME algorithms, whereas open circles indicate nodes recovered by either the NJ or the ME algorithm. Bootstrap values are shown on the nodes in percentages of 1000 replications only for values over 50%. The scale bar equals 0.020 changes per nucleotide position.

Fig. S2

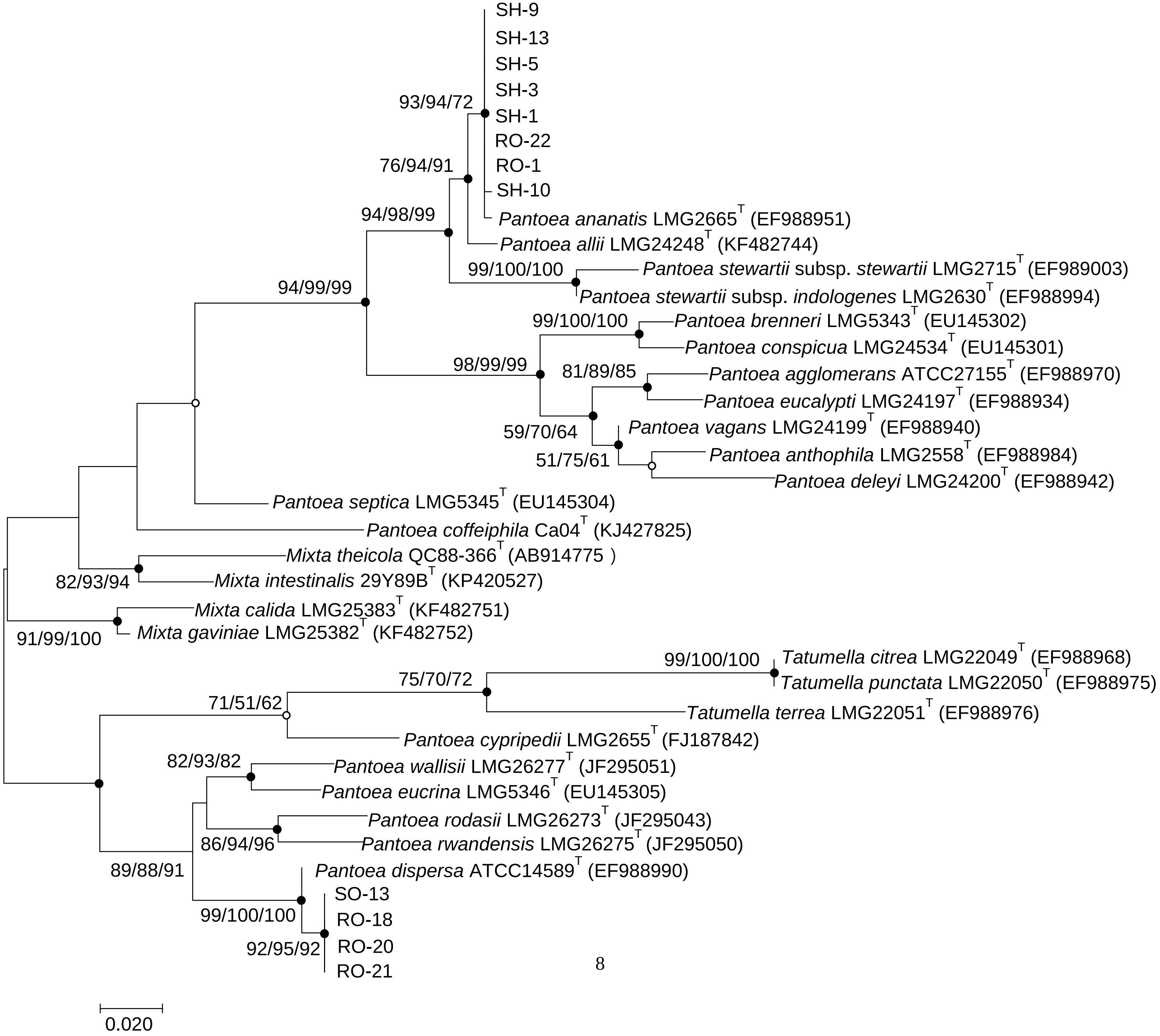
SH-9
SH-13
SH-5
SH-3
SH-1
RO-22
RO-1
SH-10
93/94/72
76/94/91
94/98/99
Pantoea ananatis LMG2665T (EF988951)
Pantoea allii LMG24248T (KF482744)
99/100/100
Pantoea stewartii subsp. stewartii LMG2715T (EF989003)
Pantoea stewartii subsp. indologenes LMG2630T (EF988994)
94/99/99
99/100/100
Pantoea brenneri LMG5343T (EU145302)
Pantoea conspicua LMG24534T (EU145301)
98/99/99
81/89/85
Pantoea agglomerans ATCC27155T (EF988970)
Pantoea eucalypti LMG24197T (EF988934)
59/70/64
Pantoea vagans LMG24199T (EF988940)
51/75/61
Pantoea anthophila LMG2558T (EF988984)
Pantoea deleyi LMG24200T (EF988942)
Pantoea septica LMG5345T (EU145304)
Pantoea coffeiphila Ca04T (KJ427825)
Mixta theicola QC88-366T (AB914775)
82/93/94
Mixta intestinalis 29Y89BT (KP420527)
Mixta calida LMG25383T (KF482751)
91/99/100
Mixta gaviniae LMG25382T (KF482752)
99/100/100
Tatumella citrea LMG22049T (EF988968)
Tatumella punctata LMG22050T (EF988975)
75/70/72
71/51/62
Tatumella terrea LMG22051T (EF988976)
Pantoea cypripedii LMG2655T (FJ187842)
82/93/82
Pantoea wallisii LMG26277T (JF295051)
Pantoea eucrina LMG5346T (EU145305)
Pantoea rodasii LMG26273T (JF295043)
86/94/96
Pantoea rwandensis LMG26275T (JF295050)
89/88/91
Pantoea dispersa ATCC14589T (EF988990)
SO-13
99/100/100
RO-18
92/95/92
RO-20
RO-21
0.020

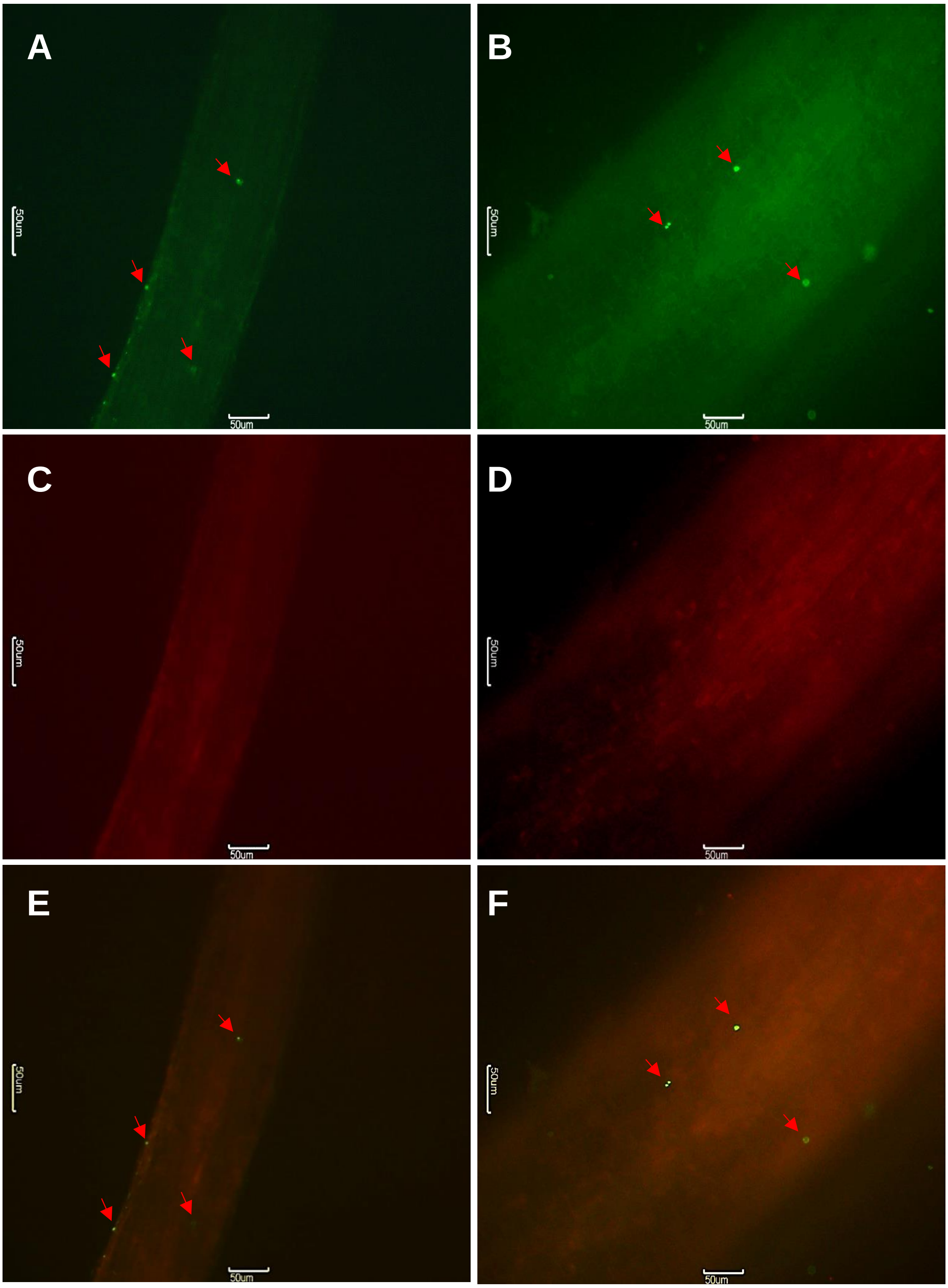

Supplementary Fig. S3. Bacterial cells of GFP-labeled RO-21 in the roots after inoculation for 18h. The images represent the micrographs of GFP filter (shown in green, **A** and **B**), autofluorescence of chlorophyll (shown in red, **C** and **D**) and the merged images from two micrographs (**E** and **F**). The red arrows point to the bacterial cells. Scale bars=50 μM.

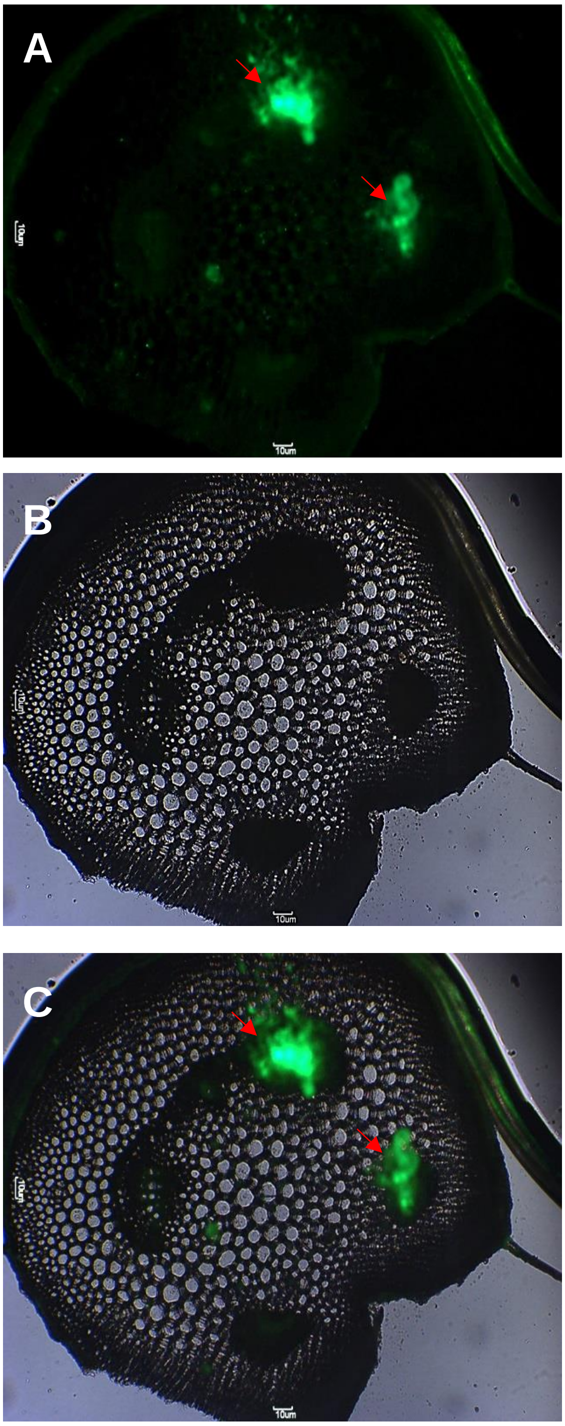

Supplementary Fig. S4. Colonization of GFP-labeled RO-21 in the sweet potato petiole tissue. The colonization of GFP-labeled RO-21 was observed in the cross-section of petiole after inoculation with GFP-labeled RO-21 for 7 d. Images were taken with the GFP filter (**A**) and bright filter (**B**), and combined image from two micrographs (**C**). The red arrows point to the bacterial aggregates. Scale bars=10 μM.

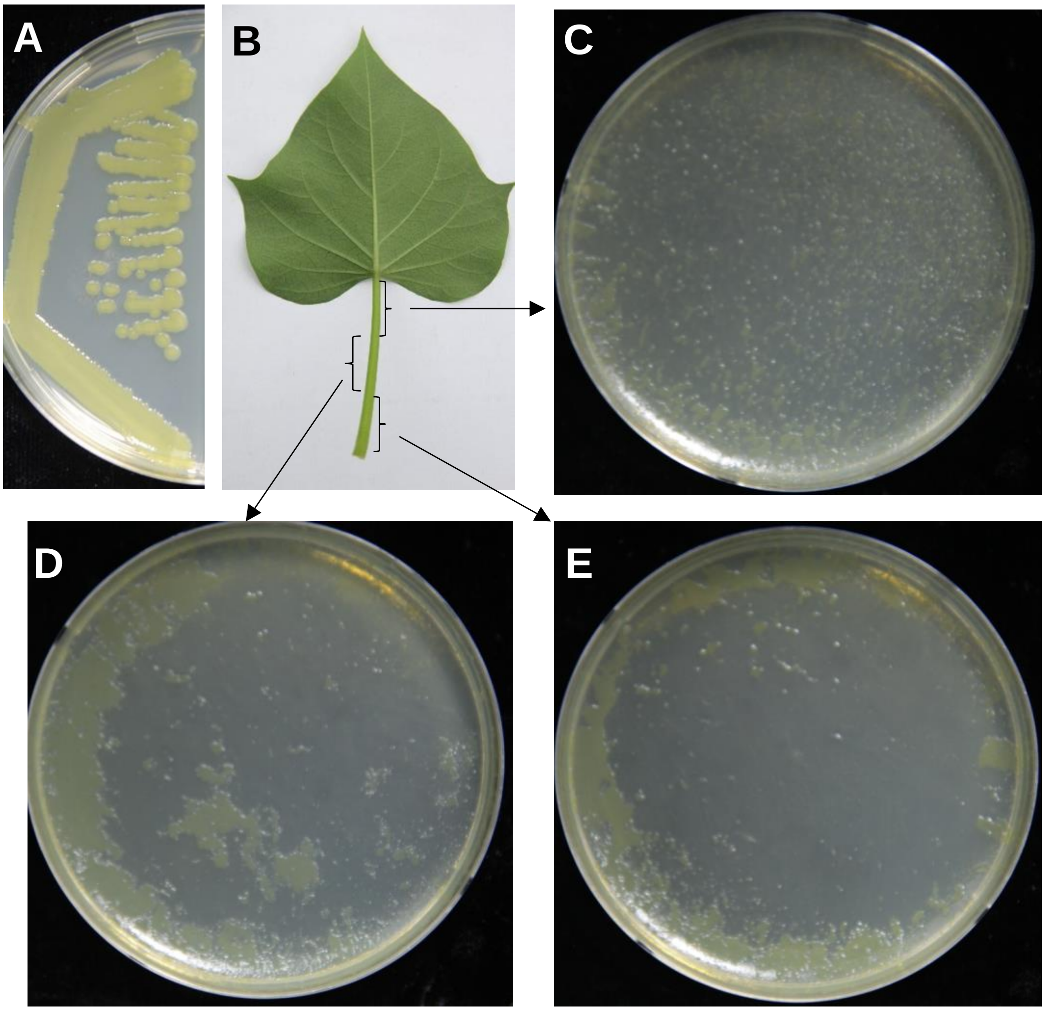

Supplementary Fig. S5. Re-isolation of GFP-labeled strain RO-21 from sweet potato plants. (**A**) The GFP-labeled strain RO-21 bacteria were streaked on selective medium. (**B**) Detached leaf petiole from sweet potato plants after inoculation with GFP-labeled RO-21 for 7 d. (**C-E**) Sweet potato leaf petioles were surface-sterilized and obtained bacterial colonies on selective medium after 3 d.

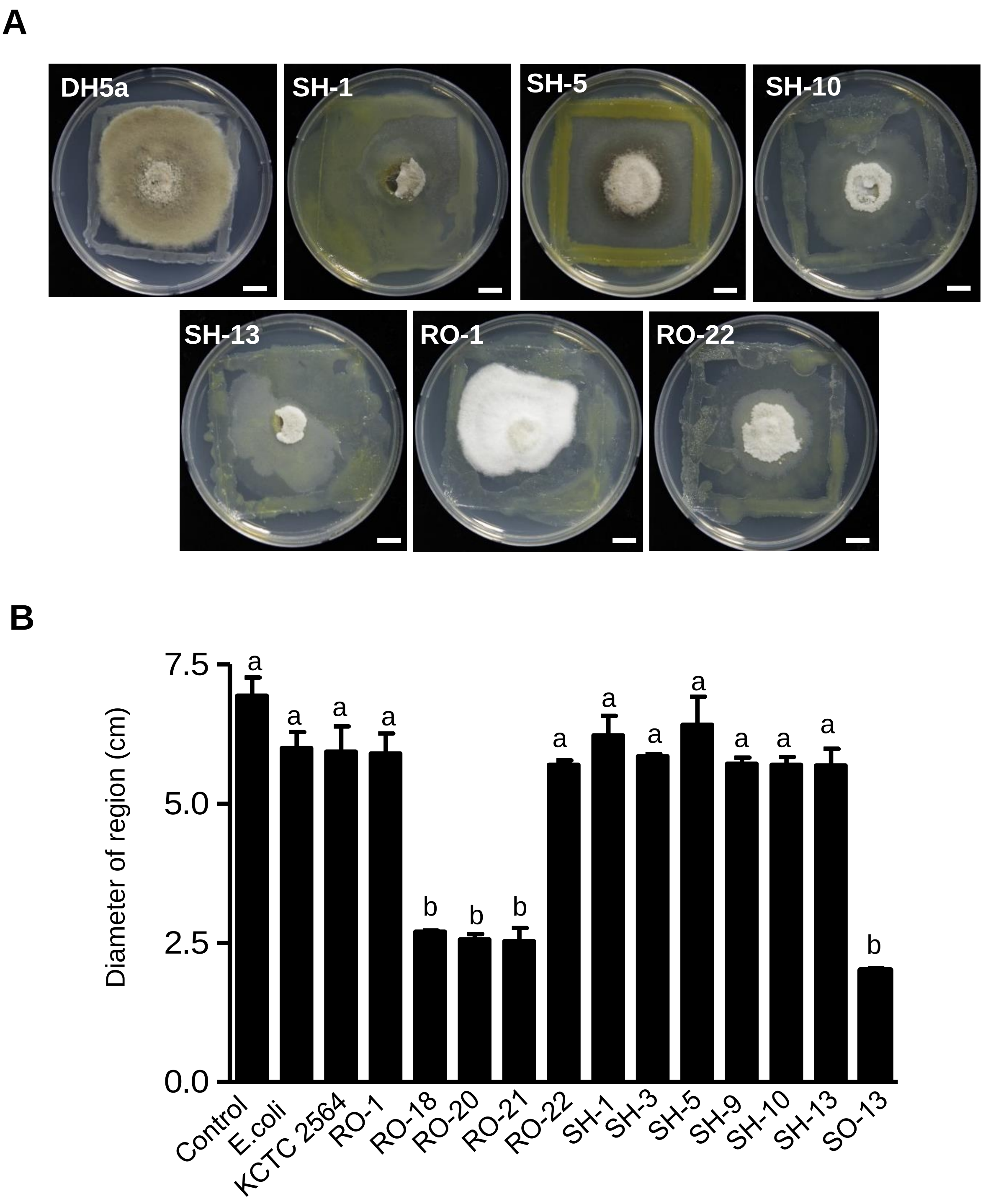

Supplementary Fig. S6. Inhibition effects of *Pantoea* isolates against the phytopathogenic fungus *C. fimbriata*. (**A**) Dual culture assay of *C. fimbriata* and *Pantoea* isolates (SH-1, SH-5, SH-10, SH-13, RO-1, and RO-22) at 16 d. *Escherichia coli* were used as controls (scale bar = 1 cm). (**B**) The diameter of *C. fimbriata* after co-incubation with *Pantoea* strains. The bars indicate the standard errors of triplicate samples. Values followed by a different letter are significantly different ($P \leq 0.05$).

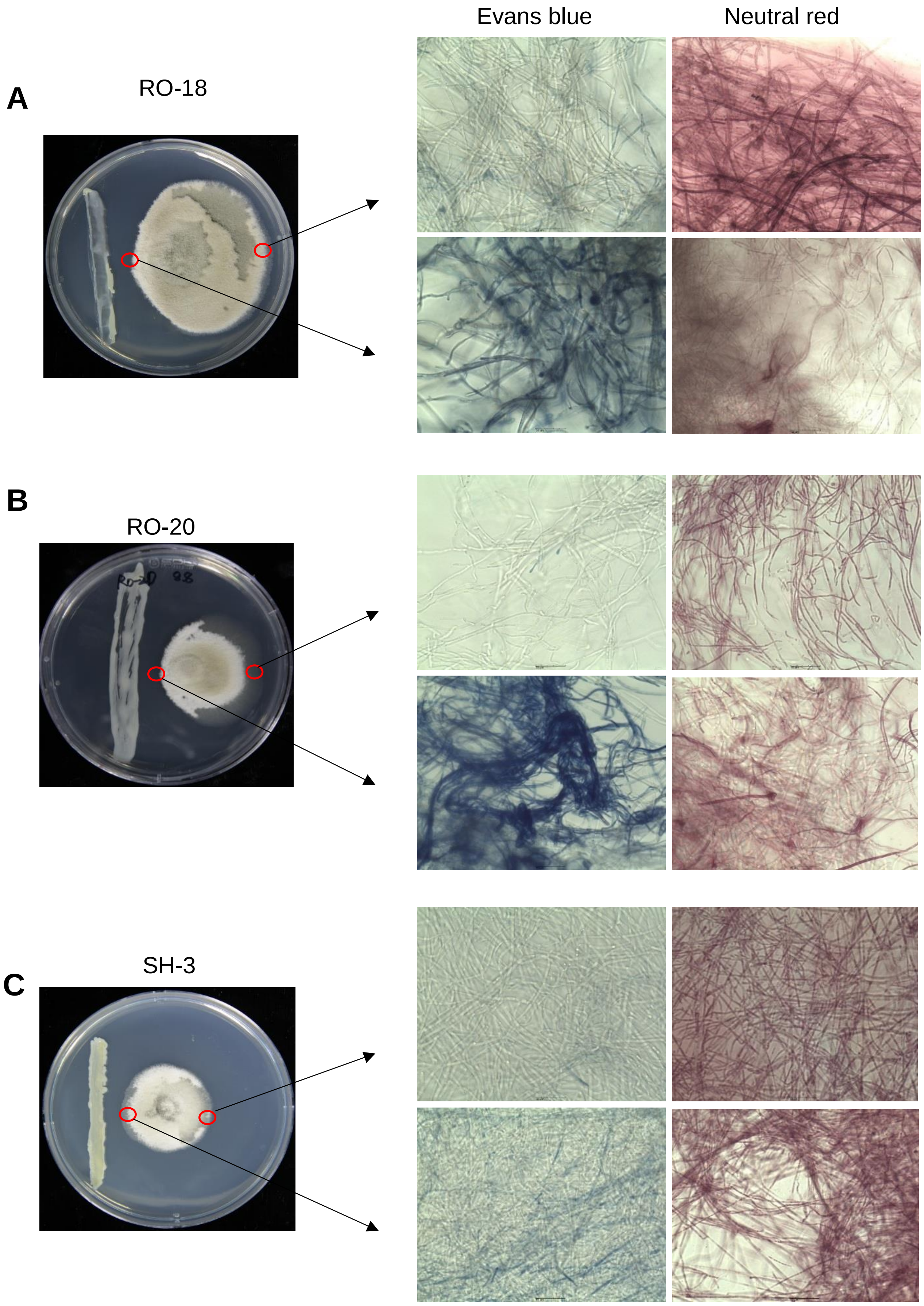
Evans blue
Neutral red
A
RO-18
B
RO-20
C
SH-3

Supplementary Fig. S7. Interaction effect of *C. fimbriata* with *Pantoea* RO-18, RO-20, and SH-3. *C. fimbriata* cells were stained with Evans blue (dead cells stain blue) and Neutral red (viable cells stain red) after co-cultivation with strain RO-18, RO-20, and SH-3 for 10 d. Mycelia growing near to RO-18 and RO-20 stained blue (**A, B**), while those in the control zone on the other side only exhibited faint staining. By contrast, mycelia that were co-cultured with SH-3 did not stain blue in any region (**C**). Scale bars are 50 μm.
